# The Spectrum projection package: improvements in estimating mortality, ART needs, PMTCT impact and uncertainty bounds

**DOI:** 10.1136/sti.2008.029868

**Published:** 2008-07-22

**Authors:** J Stover, P Johnson, B Zaba, M Zwahlen, F Dabis, R E Ekpini

**Affiliations:** 1 Futures Institute, Glastonbury, Connecticut, USA; 2 US Census Bureau, Washington, USA; 3 London School of Hygiene and Tropical Medicine, London, UK; 4 Institute of Social and Preventive Medicine, University of Bern, Bern, Switzerland; 5 Institut de Santé Publique, Epidémiologie et Développement (ISPED), Université Victor Segalen, Bordeaux, France; 6 UNICEF, New York City, USA

## Abstract

**Background:**

The approach to national and global estimates of HIV/AIDS used by UNAIDS starts with estimates of adult HIV prevalence prepared from surveillance data using either the Estimation and Projection Package (EPP) or the Workbook. Time trends of prevalence are transferred to Spectrum to estimate the consequences of the HIV/AIDS epidemic, including the number of people living with HIV, new infections, AIDS deaths, AIDS orphans, treatment needs and the impact of treatment on survival.

**Methods:**

The UNAIDS Reference Group on Estimates, Modelling and Projections regularly reviews new data and information needs and recommends updates to the methodology and assumptions used in Spectrum. The latest update to Spectrum was used in the 2007 round of global estimates.

**Results:**

Several new features have been added to Spectrum in the past two years. The structure of the population was reorganised to track populations by HIV status and treatment status. Mortality estimates were improved by the adoption of new approaches to estimating non-AIDS mortality by single age, and the use of new information on survival with HIV in non-treated cohorts and on the survival of patients on antiretroviral treatment (ART). A more detailed treatment of mother-to-child transmission of HIV now provides more prophylaxis and infant feeding options. New procedures were implemented to estimate the uncertainty around each of the key outputs.

**Conclusions:**

The latest update to the Spectrum program is intended to incorporate the latest research findings and provide new outputs needed by national and international planners.

Projections of adult HIV prevalence are prepared using the Estimation and Projection Package (EPP)^1^ for generalised epidemics and the Workbook for low-level and concentrated epidemics.^2^ The Spectrum projection package is used to determine the consequences of the prevalence projections, including the number of people living with HIV by age and sex, new infections, AIDS deaths, AIDS orphans, the need for treatment and the impact of treatment. Other demographic indicators of interest, such as life expectancy and under-five mortality are also estimated. Earlier updates to Spectrum have been described previously.^3^ A full description of the methodology is provided in the manual for the AIDS module of Spectrum (AIM).^4^ The purpose of this paper is to describe the updates to Spectrum made in the last two years and used in the 2007 global estimates of HIV/AIDS.

Spectrum is a modular program that is used to examine the consequences of current trends and future programme interventions in reproductive health. The program and manuals are updated regularly and available in multiple languages free of charge at www.FuturesInstitute.org. Model development has been funded primarily by USAID with technical collaboration from UNAIDS, WHO, UNICEF, UN Population Division, US Census Bureau, UNFPA and other organisations.

The major inputs and outputs of the AIDS module of Spectrum are shown in figure 1. Demographic projections are based on user inputs or projections prepared by the United National Population Division.^5^ The projections start with an estimate and projection of adult prevalence, which is combined with information on the age and sex distribution of prevalence and progression to death to estimate the number of new adult infections by age and sex. New infant infections are estimated from prevalence among pregnant women and the rate of mother-to-child transmission, which is dependent on infant feeding practices and the coverage of prophylaxis with antiretrovirals (ARVs). New infections progress over time to a symptomatic stage where antiretroviral treatment (ART) is needed. Those who receive first-line and or second-line ART experience extended survival. People at any stage are subject to non-AIDS mortality at the same rates as those who are not infected. Adult deaths result in orphans.

**Figure 1:**
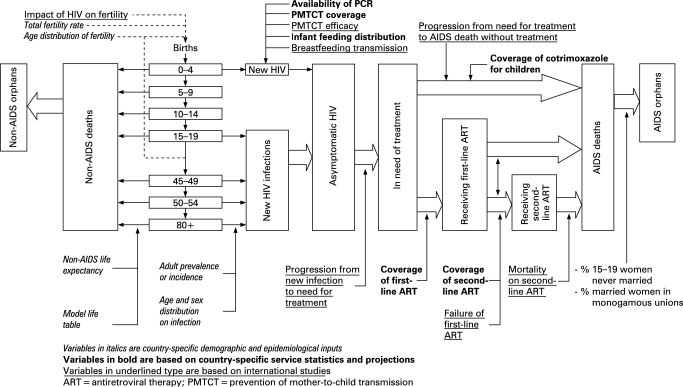
Inputs, outputs and process of the demographic and AIDS modules in Spectrum.

Several major enhancements were made for the 2007 round of estimates in response to (1) a review of the programme undertaken by the US Census Bureau, (2) new evidence from cohort studies, (3) revised child treatment guidelines, and (4) a need to produce uncertainty bounds for all estimates. The rest of this paper describes the changes to the demographic procedures for projecting by single age, a new pattern for the progression from new infection to need for treatment and AIDS death, new assumptions about survival on ART, revised prevention of mother-to-child transmission (PMTCT) treatment options and the new procedures for estimating uncertainty.

## NON-AIDS MORTALITY BY SINGLE YEAR OF AGE

Demographic projections rely on model life tables to describe the age-specific pattern of mortality corresponding to assumed levels of life expectancy at birth. The standard tables, Coale-Demeny and United Nations, show mortality in five-year age bands that are suitable for demographic projections made in five-year intervals. The rapidly changing dynamics of an AIDS epidemic require single-year projections. Previously Spectrum produced single-year projections by dividing the five-year mortality into equal amounts by single age. In order to improve the estimates of non-AIDS survival ratios by single years of age, the new Spectrum uses a modification of the Beers^6^ osculatory interpolation method to convert abridged model life-tables into complete life-tables. The Beers method has been used for many years by actuaries and demographers to interpolate curves or populations based on five-year age groups to single years of age.^7^ The problem is that the rapid change in the survival curve as a result of the relative level of infant-to-child mortality makes it difficult for a generalised procedure to reproduce that change. It has been observed that the Beers estimate of the stationary population in a life-table (L_x_) for age 1 is usually close to an independently derived value, but the problem is the relative values of the age 0 (that is, under age 1) and ages 2–4. In cases where an independent estimate of age 0 is available (as in the case of model life-tables) the Beers estimate for age 1 can be used and a polynomial can be fitted to the independent age 0, the Beers age 1, residual 2–4, and Beers 5–8, 9 and 10. This results in a smooth curve of _1_L_x_ that fits the original 0, 1–4, and 5–9 and blends smoothly to the Beers estimates for ages 10 and over. The resulting _1_L_x_ values are then used to estimate the survival ratios:

S_x_ = L_x_/L_x−1_ (1< = x< = 79)

S_0_ = L_0_/l_0_

S_80_ = T_80_/T_79_

where S_x_ = one-year survival ratio from age x − 1 to age x, for x = 0 from birth to age 0, for x = 80 from age 79+ to 80+, L_x_ = life-table stationary population age x, l_0_ = radix of life table, T_x_ = life-table stationary population ages x and above.

## PROGRESSION FROM INFECTION TO AIDS DEATHS IN THE ABSENCE OF TREATMENT

Previous versions of Spectrum relied on estimates of survival post-infection based primarily on one developing country study: the natural history cohort of the MRC/UVRI study in Masaka, Uganda^8^ (with additional data from Haiti and a Thai military cohort) implying a median survival time from infection to death of 9 years. Recent analyses produced by the Alpha network^9^ linking HIV cohort studies in a larger number of developing countries widened the evidence base for estimating survival patterns in HIV-infected adults.^10^

Comparable analyses of data from studies in six different countries^11^^–^16 showed that survival times varied by study site and age, with median survival times for those infected before age 20 more than twice as high as for those infected aged 40 and over. A meta-analysis^17^ showed that after adjusting to age 25–29 at seroconversion, median survival was longer in South African miners, 11.6 years (95% CI 9.8 to 13.7) and African population cohorts, 11.1 years (95% CI 8.7 to 14.2) than in Haiti, 8.3 years (95% CI 3.2 to 21.4) and Thailand, 7.5 years (95% CI 5.4 to 10.4). Median survival for women was about one year longer than for men, but this was explained by their younger age at infection. Before the introduction of ART, there were no discernible trends in survival time by calendar year of infection.

To make survival estimates for populations with different levels of non-HIV mortality, “net” mortality patterns were calculated in studies that also collected data on mortality patterns of those not infected with HIV.^18^ Net proportion surviving x years after infection, S_N_(x), was calculated from observed (gross) survival of infected people, S_G_(x), and the corresponding survival for uninfected people, S_U_(x), using the relation:


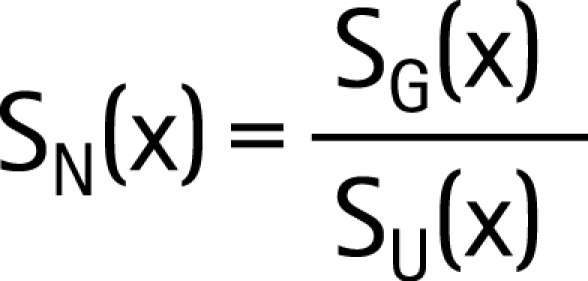


**Figure 2:**
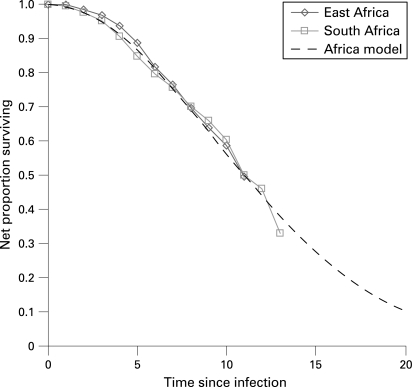
Net survival curves, for grouped studies from East Africa (population cohorts), South Africa (miners occupational cohort) and the fitted Weibull model.


Figure 2 compares “net” mortality curves for the three grouped East African community studies (Rakai and Masaka in Uganda, Kisesa in Tanzania), and for the South African miners cohort, and shows a superimposed model representation of “net” mortality among HIV-infected people, generated by the Weibull function with φ = 2 and λ = 13.21 (median 11 years):

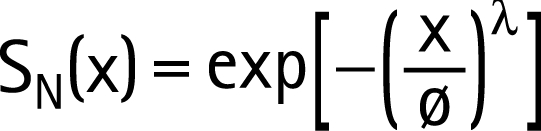


In the Spectrum model representing survival post-infection before the availability of ART, separate Weibull models are used to represent male and female survival. For most countries the male and female models have median survival times of 10.5 years and 11.5 years respectively, to allow for the older age at infection for males compared to females. For Thailand, Cambodia and Burma, the Thai cohort data are used to produce Weibull models with median survival times of 8.1 years and 8.9 years respectively.

## TIME TO ELIGIBILITY FOR ANTIRETROVIRAL TREATMENT

For a rising proportion of people living with HIV the progression to AIDS death described above is modified by antiretroviral therapy (ART). Previously eligibility for ART was estimated as occurring two years before AIDS death. A number of cohort studies provide new evidence on the time to eligibility. The eligibility of antiretroviral treatment (ART) in lower income countries (eART-linc) Collaboration is a collaboration between five cohort studies of HIV-1-infected patients from Africa^19^^–^22 and Thailand^23^ ^24^ initiated in 2007 by UNAIDS for collaborative analyses of the natural history of untreated HIV infection in lower-income countries. The eART-linc collaboration aimed at estimating duration from seroconversion to different treatment eligibility criteria, and duration from ART eligibility criteria to death from all causes. By October 2007, the eART-linc collaboration analysed 2072 seroconverters with 16 157 person-years of follow-up from five cohorts from Côte d’Ivoire,^19^ Uganda^20^^–^22 and Thailand.^23^ ^24^ Duration from seroconversion to different ART treatment eligibility criteria was estimated by fitting Weibull survival models, censoring individuals when ART start was recorded or when last seen, whichever occurred first. Consistency constraints were set so that the mean time from seroconversion to death was equal to the sum of the mean time from seroconversion to reaching ART eligibility and the mean time from ART eligibility to death. The eART-linc collaboration estimates for the median duration from ART eligibility to death were from 2.1 years to 4.0 years for different ART eligibility criteria.

The estimates of the time from ART eligibility to death (in the absence of ART) were longer than those used in earlier calculations of the number of people needing ART^25^ and were even longer when excluding the Thai studies from the analysis. The main reason for this was that the models were built in a way to incorporate the true—but never observed—time when a person first reached ART eligibility. Despite the pooling of several datasets in eART-linc, uncertainty of estimates was substantial and the available data were too scarce to estimate the relevant time periods in relation to the full ART eligibility criteria as recommended by the World Health Organization: patients who have either <200 CD4 cells ×10^6^/l or WHO stage 4 disease or a combination of 200<CD4 cells ×10^6^/l <350 and WHO stage 3. Compared to the criterion of CD4 count below 200 cells ×10^6^/l, the combined WHO criteria would result in a shorter time between seroconversion and reaching ART eligibility, and a longer period between ART eligibility and death in the absence of ART.

## SURVIVAL ON ANTIRETROVIRAL THERAPY

In the past very little information was available on the effectiveness of ART in averting death in developing countries, but the recent expansion of ART has provided new data. A literature review was conducted to update our knowledge about survival after treatment initiation^26^^–^35 (table 1). A systematic review of published reports on survival among HIV-infected patients on ART in LINCs has been conducted, focusing on those reports investigating the factors associated with mortality in adults and children. Three readers independently extracted data from articles that were selected after screening of PubMed/Medline up to December 2006 and abstracts of the 2001–6 international Conferences. Observational cohorts and clinical trials and programme reports were eligible as long as they took place in LINCs and included patients on ART. Only the studies with the occurrence of death as outcome were included in this review (the studies restricting their findings to the immunological or virological response were excluded). The details recorded included patient demographic characteristics, baseline CD4 count, survival estimate and factors associated with mortality on ART.

**Table 1:** Survival on ART, crude and adjusted for 100% mortality of patients lost to follow-up

Author, year	Country of study	Sample size	12-month survival	Loss to follow-up	Adjusted 12 months survival assuming 100% of lost to follow-up are dead	24-month survival	36-month survival	60-month survival
Braitstein 2006^26^	Africa, Asia, S America	2725	0.94	0.12	0.83			
Calmy 2006^27^	11 countries	6861	0.9	0.048	0.86			
Etard 2006^28^	Senegal	404	0.88	0.0195	0.86	0.83		0.75
Ferradini 2006^29^	Malawi	1308	0.81	0.0385	0.78	0.72		
Munderi 2006^30^	Uganda	1015				0.94		
Bourgeois 2005^31^	Cameroon	109	0.92	0.028	0.89			
Laurent 2005^32^	Dakar	176				0.84	0.81	
Lawn 2005^33^	South Africa	578	0.93	0.071	0.86			
Severe 2005^34^	Haiti	910	0.82	0.078	0.76			
Coetzee 2004^35^	South Africa	287	0.86	0.007	0.85	0.86		
								
Median survival			0.89		0.86	0.84		
Weighted average					0.84			
Standard deviation			0.05			0.08		

For adult patients, a total of 25 articles were included in this review among 878 screened. Twelve months after starting ART, the overall mortality rate ranged from 0.06 to 0.26—that is, the 12-month probability of survival ranged from 0.74 to 0.94. It was generally lower in patients starting ART with CD4 <50 cells ×10^6^/l (range 0.67–0.91 ×10^6^/l). The factors associated with poorer survival among adults were low CD4 count (15 reports out of 15 studying this factor), advanced WHO stage 3 or 4 (10/12 reports), low body mass index (6/8 reports), low haemoglobinaemia, with different cut-off values (5/6 reports), male gender (3/7 reports) and poor adherence (1/1 report). The death rate was the highest in the first three months following the initiation of ART (from 5.1 to 37.3 per 100 person-years among five reports). For children, only three articles could be included among 23 originally selected. The six-month survival after ART initiation varied from 0.91 to 0.95. A low CD4 percentage was associated with poorer survival on ART. Little or no data were available on survival according to the different first-line regimens and the treatment switches occurring in the first year on ART.

**Figure 3:**
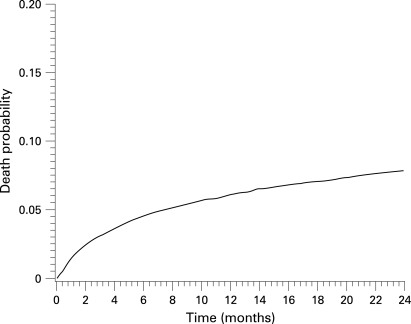
Probability of death among 36 615 adult ART-treated patients in 17 cohorts in Africa, Asia and South America (courtesy of ART-LINC Collaboration).

ART treatment programmes in LINCs have survival rates comparable to those reported in developed countries in the early phase of ART introduction 10 years ago.^26^ The most recent survival estimates of the ART-LINC Collaboration, pooling individual adult data from 36 615 patients on ART in 17 cohorts in Africa, Asia and South America, are 0.939 one year after starting treatment (95% confidence interval: 0.936 to 0.942) and 0.929 after two years (0.926 to 0.932) (fig 3). However, the frequency of patients lost to follow-up is of serious concern to fully interpret the survival/mortality findings in the cohorts reporting both events^36^ (table 1). In the ART-LINC Cohort Collaboration, the proportion of patients lost to follow-up in the first six months on ART was as high as 16% among 7651 adults enrolled in 15 cohorts.^37^ In Spectrum we have used values of 0.85 for first-year adult survival on ART (rounded weighted average from table 1). Data on survival in subsequent years are sparse but are clearly much higher than first-year survival. We have used 0.95 as the annual survival for subsequent years. The annual survival for children over age 1 year on ART is set at 90% in the first year of ART and 95% in subsequent years, based on treatment results in children in Abidjan.^38^ ^39^ Survival on ART among children under age 1 year is assumed to be 80%.

## PROGRAMMES TO PREVENT MOTHER-TO-CHILD TRANSMISSION OF HIV

Recent scientific and programmatic evidence has led to the review of approaches to the use of ARV drugs in the context of PMTCT. In general, longer prophylactic ARV regimens starting earlier in pregnancy are more efficacious than shorter regimens. Studies conducted in both Asia and sub-Saharan Africa have shown that a combination regimen of AZT plus single-dose nevirapine (SD-NVP) is more effective than single-drug regimens in formula-fed as well as breastfeeding populations. AZT plus SD-NVP is as effective as more complex regimens of AZT/3TC starting at around 32-36 weeks given alone or plus SD-NVP. In the absence of any intervention, postnatal transmission of HIV accounts for 5% to 20% additional transmission rate. Mixed feeding is associated with higher transmissions rates compared with both replacement feeding and exclusive breast feeding. Three major cohort studies conducted in Côte d’Ivoire, South Africa and Zimbabwe have shown that exclusive breast feeding for up to six months is associated with a threefold to fourfold decreased risk of postnatal transmission. While data exist on the impact of single and dual prophylactic regimens on postpartum transmission through breast feeding, the safety and efficacy of ART on this mode of transmission is still under investigation. However, programme data from Botswana, Cote d’Ivoire, Mozambique and Uganda indicate that ART initiated in pregnant women might reduce postnatal transmission through breast feeding.

Concerns have been raised regarding the risk of decreased virological response in women with viral resistance related to intrapartum exposure to SD-NVP. Recent data showed that an AZT/3TC “tail” given at the time of SD-NVP and for a short time post partum reduces viral drug resistance when SD-NVP is given either alone or in combination with AZT.

WHO recommends ART for all pregnant women who are eligible for treatment. The goal is to secure the health of women, significantly reduce HIV transmission to their infants and improve child wellbeing and survival. For pregnant women with HIV who do not yet require ART, antiretroviral prophylactic regimens are recommended for the prevention of MTCT. The recommended regimens are based around AZT (from 28 weeks of pregnancy or as soon as possible thereafter) plus single-dose nevirapine, plus maternal 7-day “tail” of AZT and 3TC and one-week AZT for the infant.

Various ARV regimens for PMTCT are currently implemented in low-income and middle-income countries. These regimens range from single drugs, such as SD-NVP and short-course AZT, to dual and triple drugs. Spectrum has been updated to include the full range of treatment and prophylactic options now available and the distribution and duration of infant feeding methods as shown in table 2.

**Table 2:** Probability of mother-to-child transmission of HIV (%)

Duration of breast feeding in the general population	Treatment	Mixed breast feeding	Replacement feeding	Exclusive breast feeding
Approximately 6 months	None	26	20	23
	Single-dose nevirapine	17	11	14
	Dual-prevention ARV	10	4	7
	Triple-treatment ARV	4	2	3
Approximately 12 months	None	30.5		
	Single-dose nevirapine	21.5		
	Dual-prevention ARV	14.5		
	Triple-treatment ARV	5		
Approximately 18 months	None	35		
	Single-dose nevirapine	26		
	Dual-prevention ARV	19		
	Triple-treatment ARV	6		

**Mixed breast feeding: in addition to replacement feeding rates**

*First six months:* 1% per month for first 6 months (total 6%) (based on reports by Iliff *et al*^45^ and Rollins^46^: approximately double the hazard of exclusive breastfeeding).

*Thereafter:* 0.75% per month for next 6 months (total 4.5%) and for next 6 months (total 4.5%) (based on BHITS meta-analysis^47^: 8.9% per 100 person-years).

*Triple-treatment ARV:* in absence of data, assume 2% additional transmission for first 6 months, then 1% for next 6 months and again 1% for next 6 months.

**Replacement feeding**

*No treatment:* 20%: based on 2004 UNICEF/UNAIDS/WHO/UNFPA review.^40^

*Single-dose nevirapine:* based on data from Jackson *et al* (11.8% at 6–8 weeks in breastfeeding population)^41^ and Moodley *et al* (10.7% among replacement feeding population).^42^

*Dual-prevention ARV:* based on Ditrame (6.5% at 6 weeks (with some breastfeeding transmission))^43^ and PPHT-2 (1.9–2.8% among replacement feeding population).^44^

*Triple-treatment ARV:* based on western studies quoted in report by UNICEF/UNAIDS/WHO/UNFPA.^40^

**Exclusive breast feeding**

*In addition to replacement breastfeeding rates:* 0.5% additional transmission per month (total 3%) (based on reports by Iliff *et al*^45^ and Rollins^46^).

*Triple-treatment ARV:* in absence of data, assume 1% additional transmission.

## UNCERTAINTY ANALYSIS

Spectrum produces a point estimate for each indicator for each year. However, there may be a considerable amount of uncertainty associated with each point estimate owing to: (1) uncertainty around the prevalence curve produced by EPP or the Workbook, and (2) uncertainty around input assumptions that are based on studies from population samples in selected countries. In previous rounds uncertainty bounds were estimated by cumulating the uncertainty for each step in the calculation for a typical generalised or concentrated epidemic and applying the resulting bounds to all countries. In 2005 those calculations were done for a large number of countries in each category. For the 2007 estimates these procedures have been added to Spectrum and are applied to estimate uncertainty bounds for each country.

The prevalence trend is an input to Spectrum that comes from EPP for generalised epidemics and from the Workbook for low-level and concentrated epidemics as described in other articles in this supplement. EPP generates 1000 prevalence curves from its Bayesian melding approach and passes those to Spectrum for use in this analysis. The Workbook method passes just a series of annual estimates of adult prevalence. Spectrum generates 1000 prevalence curves from the annual estimates of prevalence by fitting a single logistic curve for countries where prevalence is rising or stable and a double logistic curve if prevalence is falling. The equation for the single logistic curve is:

Prev_t_ = a e^α (t−t0)^/(1+e^α (t−t0)^)

For the double logistic curve the equation is:

Prev_t_ = α e^(t−t0)^/(1+α e^(t−t0)^)×[2 a e^−β(t−t0)^/(1+e^−β(t−y0)^)+b]

where a = parameter describing the asymptote, α = parameter describing the rate of increase, t0 = year the curve reaches one-half of the asymptote, b = asymptote after the decline, β = rate of decline.

For each of 1000 iterations the prevalence points are randomly varied within some range and a logistic curve is fit to the resulting points. The range of variation around each data point is dependent on the quality of the surveillance used to estimate prevalence. For countries with good surveillance systems the range is −40% to +60% of the point estimate. The range is increased to −48% and +102% for countries with medium quality surveillance and to −53% to +183% for those with poor quality surveillance systems. The quality of national surveillance systems has been estimated on the basis of the frequency and consistency of surveillance and the number of urban and rural sites.^48^

The Spectrum uncertainty analysis consists of a large number of Monte Carlo runs, usually 1000, that each randomly select a prevalence curve from the EPP or Workbook fits and input values for other parameters from a range that can be set by the user. The default ranges use standard deviations of 0.05 for mother-to-child transmission rates, 0.2 for the effects of HIV on fertility for 15–19 year old women, 0.1 for the effects of HIV on fertility among 20–49 year old women, 0.10 for the effect of cotrimoxazole on mortality and 0.05 for the ratio of female to male prevalence. Ranges for the age distribution of prevalence are estimated from national surveys of HIV prevalence in sub-Saharan Africa. Ranges for the progression from infection to need for treatment and need for treatment to death are generated from Weibull curves that vary the median time of progression by plus or minus two years. The user has the opportunity to examine and change any of these default ranges before processing. For example, the uncertainty around the female-to-male prevalence ratio may be low in a country that has recently had a national sero-survey but would be considerably higher in a low-level or concentrated epidemic without such a survey. Not all of these inputs will affect the final results. For example, if cotrimoxazole is not used in a particular country then variations in the assumption of effectiveness of cotrimoxazole will not affect the estimate of child AIDS deaths. Once processing starts Spectrum generates 1000 different runs randomly selecting values from these ranges. The results are stored and then sorted to determine the ranges for any percentile selected. We term these ranges plausibility bounds to indicate that they are not true uncertainty ranges.

## OTHER ENHANCEMENTS

There are a number of other updates to Spectrum for the 2007 estimates. All the demographic inputs have been updated to use the 2006 round of estimates and projections from the United National Population Division.^5^ There are new utilities to ease the manipulation of a large number of country files. These utilities include Extract, which extracts the time series for any indicator or selection of indicators from multiple Spectrum projection files; Aggregate, which combines multiple projection files into a single file for a region; and Scenario Generator, which applies the same set of scenario projection assumptions for the key HIV inputs across a selection of projection files.

Another new feature allows the input of incidence, rather than prevalence, to drive the Spectrum projection. This is particularly useful in examining the impact of prevention interventions when the impact on incidence can be determined or for examining the impact on prevalence of enhanced survival due to ART.

## STRENGTHS

The outputs of Spectrum include indicators useful for understanding the scope and magnitude of the epidemic, such as numbers of people infected, new infections and AIDS deaths, and indicators needed for planning the response, such as the number of people needing treatment or PMTCT services. The estimates are used by both national programmes and by international agencies mobilising commitment and resources. Recent updates to Spectrum incorporate the latest research findings and allow national programmes to examine the implications of those results for their programmes. Key researchers contribute their expertise to the selection of approaches and assumptions used in Spectrum through participation in meetings of the UNAIDS Reference Group on Estimates, Modelling and Projections.

## LIMITATIONS

Many of the assumptions in Spectrum are derived from a small number of studies. These studies may not be representative of all populations. Many more studies are generally available for African populations than for those in other regions. The results from research studies with good service delivery and adherence and may not reflect the impact that will be achieved when implemented nationally. The new procedures to estimate uncertainty rely on expert judgment and the variation found within available studies and surveys. This may underestimate the uncertainty caused by applying these assumptions to other regions.

## CONCLUSIONS

Spectrum is one of a package of tools that has been developed to estimate the impact of the AIDS epidemic at the national level. It continues to be updated to incorporate the latest research findings and provide indicators needed for programme planning. Recent updates of the progression from infection to death in the absence of treatment have benefited from the experience of long-running cohort studies and newer treatment cohorts have provided valuable data on the effects of ART on survival. These updates allow analysts from national programmes to estimate their needs in order to achieve national goals for prevention and treatment.

**Key messages**

The Spectrum software is used to calculate HIV indicators of interest on the basis of previously prepared prevalence estimates. In the last two years new calculations and assumptions have been added in order to incorporate the latest data and respond to the international commitment for expanded treatment for adults and children.

## References

[1] Brown T Salomon JA Alkema L Progress and challenges in modelling country-level HIV/AIDS epidemics: the UNAIDS Estimation and Projection Package 2007. Sex Transm Infect 2008;84 Suppl 1:i5–i10 18647867 10.1136/sti.2008.030437 PMC2569145

[2] Lyerla R Gouws E Garcia-Calleja JM The 2005 Workbook: an improved tool for estimating HIV prevalence in countries with low level and concentrated epidemics. Sex Transm Infect 2006;82 (Suppl 3):iii41–4 16735292 10.1136/sti.2006.020198 PMC2576736

[3] Stover J Walker N Grassly NC Projecting the demographic impact of AIDS and the number of people in need of treatment: updates to the Spectrum projection package. Sex Transm Infect 2006;82 Suppl 3:iii45–iii50 16735293 10.1136/sti.2006.020172 PMC2576732

[4] Stover J AIM: a computer program for making HIV/AIDS projections and examining the social and economic impact of AIDS. Glastonbury, CT: Futures Institute, December 2007 Available at www.FuturesInstitute.org

[5] United Nations Population Division World population prospects: the 2006 revision. POP/DB/WPP/Rev.2006/1/F1, May 2007

[6] Beers H Discussion of papers presented in the record, no 68: ‘Six-term formulas for routine actuarial interpolation,’ by Henry S. Beers. Record of the Institute of Actuaries 1945;34 Part I69:59–60

[7] Swanson D Siegel J Methods and materials of demography. New York: Academic Press, 204;728

[8] Morgan D Whitworth JAG The natural history of HIV-1 infection in Africa. Nat Med 2001;7:143–5 11175832 10.1038/84564

[9] http://www.lshtm.ac.uk/cps/alpha/ (accessed 2 Dec 2007)

[10] Ghys P Zaba B Prins M Survival and mortality of people infected with HIV in low- and middle income countries—results from the extended ALPHA network. (Editorial introduction) *AIDS* 2007;21 (suppl 6):S1–4 10.1097/01.aids.0000299404.99033.bf PMC5786260 18032932

[11] Isingo R Zaba Marston M Survival after HIV infection in the pre-antiretroviral therapy era in a rural Tanzanian cohort. AIDS 2007;21 (suppl 6):S15–9 18032939 10.1097/01.aids.0000299405.06658.a8 PMC5784802

[12] Lutalo T Gray R Wawer M Survival of HIV-infected treatment-naive individuals with documented dates of seroconversion in Rakai, Uganda. AIDS 2007;21 (suppl 6):S21–9 10.1097/01.aids.0000299406.44775.de 18032934

[13] Van der Paal L Shafer LA Todd J HIV-1 disease progression and mortality before the introduction of highly active antiretroviral therapy in rural Uganda. AIDS 2007;21 (suppl 6):S31–7 10.1097/01.aids.0000299407.52399.05 18032935

[14] Peters P Karita E Kayitenkore K HIV-infected Rwandan women have a high frequency of long-term survival. AIDS 2007;21 (suppl 6):S39–46 10.1097/01.aids.0000299408.52399.e1 18032936

[15] Rangsin R Piyaraj P Sirisanthana T The natural history of HIV-1 subtype E infection in young men in Thailand with up to 14 years of follow-up. AIDS 2007;21 (suppl 6):S47–54 10.1097/01.aids.0000299409.29528.23 18032937

[16] Nelson KE Costello C Suriyanon V Survival of blood donors and their spouses with HIV-1 subtype E (CRF01 A_E) infection in northern Thailand, 1992–2007. AIDS 21 (suppl 6):S55–63 18032938 10.1097/01.aids.0000299410.37152.17

[17] Todd J Glynn J Marston M Time from HIV sero-conversion to death prior to ART: a collaborative analysis of eight studies in six developing countries. AIDS 2007;21 (suppl 6):S55–63 18032940 10.1097/01.aids.0000299411.75269.e8 PMC5784803

[18] Marston M Todd J Glynn JR Estimating ‘net’ HIV-related mortality and the importance of background mortality rates. AIDS 2007;21 (suppl 6):S65–71. 18032941 10.1097/01.aids.0000299412.82893.62 PMC5786261

[19] Minga A Danel C Abo Y Progression to WHO criteria for antiretroviral therapy in a 7-year cohort of adult HIV-1 seroconverters in Abidjan, Cote d’Ivoire. Bull World Health Organ 2007;85:116–23 17308732 10.2471/BLT.06.032292 PMC2636271

[20] Morgan D Maude GH Malamba SS HIV-1 disease progression and AIDS-defining disorders in rural Uganda. Lancet 1997;350:245–50 9242801 10.1016/S0140-6736(97)01474-8

[21] Sewankambo NK Gray RH Ahmad S Mortality associated with HIV infection in rural Rakai District, Uganda. AIDS 2000;14:2391–400 11089628 10.1097/00002030-200010200-00021

[22] Wawer MJ Serwadda D Gray RH Trends in HIV-1 prevalence may not reflect trends in incidence in mature epidemics: data from the Rakai population-based cohort, Uganda. AIDS 1997;11:1023–30 9223737 10.1097/00002030-199708000-00011

[23] Rangsin R Chiu J Khamboonruang C The natural history of HIV-1 infection in young Thai men after seroconversion. J Acquir Immune Defic Syndr 2004;36:622–9 15097306 10.1097/00126334-200405010-00011

[24] Nagachinta T Duerr A Suriyanon V Risk factors for HIV-1 transmission from HIV-seropositive male blood donors to their regular female partners in northern Thailand. AIDS 1997;11:1765–72 9386812 10.1097/00002030-199714000-00014

[25] Boerma JT Stanecki KA Newell ML Monitoring the scale-up of antiretroviral therapy programs: methods to estimate coverage. Bull World Health Organ 2006;84:145–50 16501733 10.2471/blt.05.025189 PMC2626537

[26] Braitstein P Brinkhof MW Dabis F Mortality of HIV-1-infected patients in the first year of antiretroviral therapy: comparison between low-income and high-income countries. Lancet 2006;367:817–24 16530575 10.1016/S0140-6736(06)68337-2

[27] Calmy A Pinoges L Szumilin E Generic fixed-dose combination antiretroviral treatment in resource-poor settings: multicentric observational cohort. AIDS 2006;20:1163–9 16691068 10.1097/01.aids.0000226957.79847.d6

[28] Etard JF Ndiaye I Thierry-Mieg M Mortality and causes of death in adults receiving highly active antiretroviral therapy in Senegal: a 7-year cohort study. AIDS 2006;20:1181–9 16691070 10.1097/01.aids.0000226959.87471.01

[29] Ferradini L Jeannin A Pinoges L Scaling up of highly active antiretroviral therapy in a rural district of Malawi: an effectiveness assessment. Lancet 2006;367:1335–42 16631912 10.1016/S0140-6736(06)68580-2

[30] Munderi P Watera C . Survival and causes of death, 2 years after introduction of Antiretroviral Therapy in Africa: a historical cohort comparison in Entebbe, Uganda. XVI International AIDS Conference, Toronto, Toronto, Canada: 2006

[31] Bourgeois A Laurent C Mougnutou R Field assessment of generic antiretroviral drugs: a prospective cohort study in Cameroon. Antivir Ther 2005;10:335–41 15865228

[32] Laurent C Ngom Gueye NF Ndour CT Long-term benefits of highly active antiretroviral therapy in Senegalese HIV-1-infected adults. J Acquir Immune Defic Syndr 2005;38:14–7 15608518 10.1097/00126334-200501010-00003

[33] Lawn SD Myer L Harling G Determinants of mortality and nondeath losses from an antiretroviral treatment service in South Africa: implications for program evaluation. Clin Infect Dis 2006;43:770–6 16912954 10.1086/507095

[34] Severe P Leger P Charles M Antiretroviral therapy in a thousand patients with AIDS in Haiti. N Engl J Med 2005;353:2325–34 16319381 10.1056/NEJMoa051908

[35] Coetzee D Hildebrand K Boulle A Outcomes after two years of providing antiretroviral treatment in Khayelitsha, South Africa. AIDS 2004;18:887–95 15060436 10.1097/00002030-200404090-00006

[36] Dalal RP Macphail C Mghayi M Characteristics and outcomes of adult patients lost to follow-up at an antiretroviral treatment clinic in Johannesburg, South Africa. J Acquir Immune Defic Syndr 2008;47:101–7 17971708 10.1097/QAI.0b013e31815b833a

[37] Brinkhof MWG Dabis F Myer L Early loss to program in HIV-infected patients starting potent antiretroviral therapy in lower-income countries. Bull World Health Organ (in press). 10.2471/BLT.07.044248 PMC2647487 18670668

[38] Fassinou P Elenga N Rouet F Highly active antiretroviral therapies among HIV-1-infected children in Abidjan, Cote d’Ivoire. AIDS 2004;18:1905–13 15353976 10.1097/00002030-200409240-00006

[39] Rouet F Fassinou P Inwoley A Long-term survival and immuno-viroloigical response of African HIV-1-infected children to highly active antiretroviral therapy regimens. AIDS 2006;20:2315–19 17117017 10.1097/QAD.0b013e328010943b

[40] UNICEF/UNAIDS/WHO/UNFPA HIV transmission through breastfeeding. A review of available evidence.

[41] Jackson JB Musoke P Fleming T Intrapartum and neonatal single-dose nevirapine compared with zidovudine for prevention of mother-to-child transmission of HIV-1 in Kampala, Uganda: 18-month follow-up of the HIVNET 012 randomised trial. Lancet 2003;362:859–68 13678973 10.1016/S0140-6736(03)14341-3

[42] Moodley D Moodley J Coovadia H A multicenter randomized controlled trial of nevirapine versus a combination of zidovudine and lamivudine to reduce intrapartum and early postpartum mother-to-child transmission of human immunodeficiency virus type 1. J Infect Dis 2003;187:725–35 12599045 10.1086/367898

[43] ANRS 1201/1202 DITRAME PLUS Study Group Field efficacy of zidovudine, lamivudine and single-dose nevirapine to prevent peripartum HIV transmission. AIDS 2005;19:309–18 15718842 PMC2474891

[44] Lallement M Jourdain G Le Coeur S Single-dose perinatal nevirapine plus standard zidovudine to prevent mother-to-child transmission of HIV-1 in Thailand. N Engl J Med 2004;351:217–28 15247338 10.1056/NEJMoa033500

[45] Iliff PI Piwoz EG Tavenqwa NV Early exclusive breastfeeding reduces the risk of postnatal HIV-1 transmission and increases HIV-free survival. AIDS 2005;19:699–708 15821396 10.1097/01.aids.0000166093.16446.c9

[46] Rollins N HIV transmission and mortality associated with exclusive breastfeeding: implications for counselling HIV-infected women. Presentation. http://www.path.org/files/Nigel_Rollins.pdf (Accessed 26 December 2006.)

[47] The Breastfeeding and HIV International Transmission Study Group Late postnatal transmission of HIV-1 in breast-fed children: an individual patient data meta-analysis. J Infect Dis 2004;189:2154–66 15181561 10.1086/420834

[48] Garcia-Calleja JM Zabiewski E Gys PD A global analysis of trends in the quality of HIV sero-surveillance. Sex Transm Infect 80 (Suppl 1):i25–30 15249696 10.1136/sti.2004.010298 PMC1765843

